# Recent development of eco-friendly nanocomposite carbon paste electrode for voltammetric determination of Cd(II) in real samples

**DOI:** 10.1007/s44211-022-00214-3

**Published:** 2022-11-19

**Authors:** Asmaa M. Abdel Rahim, Esraa M. M. Mahmoud

**Affiliations:** Chemistry Department, Faculty of Science, Minia University, Minia, 61511 Egypt

**Keywords:** Mango seed kernel, Nanocomposite, Eco-friendly adsorbent, Graphite reinforcement carbon paste electrode

## Abstract

Using eco-friendly, cheap, and available adsorbents is promising for the determination of metal ions. So, this study focuses on the modification of graphite reinforcement carbon paste electrode (GRCPE) with mango seed kernel (MSK) for voltammetric determination of Cd(II). Moreover, to increase the surface area of this adsorbent, it was prepared in nanosized that formed nanoparticles of mango seed kernel (MSK-NPs). The developed nanocomposite electrode of carbon paste electrode modified with nanoparticles of mango seed kernel (MSK-NPs@GRCPE) was characterized using Fourier transform infrared (FTIR) and Scanning electron microscopy (SEM). The effect of pH, buffer solution, and supporting electrolyte as experimental conditions were optimized through differential pulse adsorptive anodic stripping voltammetric method (DPA_d_ASV). Britton-Robinson buffer pH = 3.9 at *E*_acc_ = – 1400 mV, *t*_acc_ = 30 s, pulse width = 10 ms and sampling time = 8 ms were the optimum conditions for determination of Cd(II). The LOD and LOQ of MSK-NPs@GRCPE were calculated at 5.44 × 10^–9^ and 1.65 × 10^–8^ M, respectively. Compared with bare graphite reinforcement carbon paste electrode (BGRCPE), the nanocomposite MSK-NPs@GRCPE has a lower detection limit, indicating that the presence of MSK-NPs could greatly improve the response to Cd(II). The practical applicability of the electrode was verified by the determination of Cd(II) in chocolate and white rice samples. The results show high selectivity and sensitivity for Cd(II) in real samples.

**Graphical abstract:**

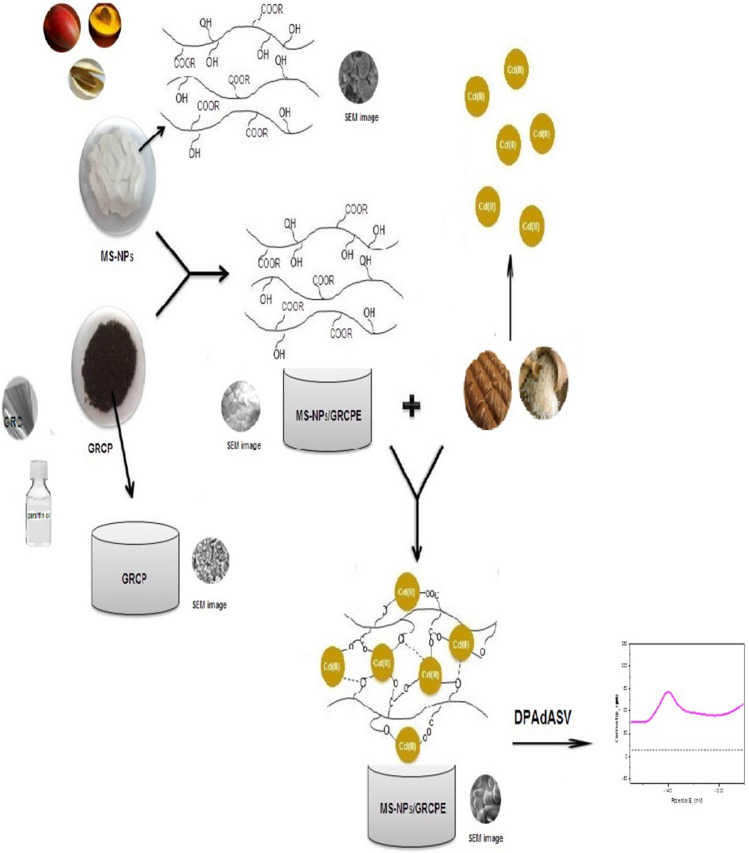

## Introduction

Recently, cheaper and more effective adsorbents can be formed from many natural waste materials from industrial and agricultural activities. Agricultural waste residues are economical and environmentally friendly due to their chemical composition, availability, renewable nature, and low cost and are viable options for the adsorption and determination of many metal ions in their samples. The primary components of these waste materials include hemicellulose, lignin, lipids, proteins, simple sugars, water, hydrocarbons, starch, and contain a variety of functional groups [1]. Among these agricultural waste materials, mango seed kernel which rich in functional groups that have a high metal-binding capability. This kernel contains most of the essential amino acids such as leucine and valine and is a rich source of gallic acid [2] that can be complexed with metal ions [1].

On the other hand, to increase the efficiency of adsorbent recent researches have turned to the use of nanoparticles due to their large specific surface area that can reveal a wide variety of interactions, depending on their nature, with target analytes. Furthermore, the nanometric dimension provides the material with new physicochemical properties and small internal diffusion resistance [3] which can be very effective for adsorbing heavy metals like Cd(II).

Cd(II) as one of these heavy metal ions reaches the food chain through various geogenic and anthropogenic activities. Excessive cadmium consumption poses a significant threat to public health due to its toxicity which is represented in causing gastrointestinal pain, nausea, softening of bones, and kidney damage. Accumulation of cadmium within the kidneys can eventually cause renal failure [4, 5]. Maximum levels of cadmium in foodstuffs are currently between 0.05 and 0.2 mg kg^−1^ wet weight [6]. There are many sources for Cd(II) in real samples between these samples are chocolate and rice. Chocolate and chocolate-based candies are common treats, snakes, or gifts for children and adults alike. Coca, milk, and fats, which are the main ingredients in chocolate, each of them considered potential source of cadmium. Thus, the determination of Cd(II) levels in chocolate is an imperative issue for manufacturers and chocolate consumers around the world. The second real sample which contains cd(II) is rice. Cadmium (Cd), lead (Pb), and arsenic (As) are the main metals, which are of toxicological importance, present in Egyptian white rice [7]. The importance of Egyptian white rice was emphasized by the UN General Assembly declared the year 2004 the International “Year of Egyptian white rice, under the slogan Egyptian white rice is life” [8]. Thus, veritable growing attention has been directed toward the content of environmental contaminates in Egyptian white rice. The FAO/WHO Joint Expert Committee (JECFA) established a provisional tolerable weekly intake (PTWI) for Cd(II) of 0.007 mg kg^−1^bodyweight, based on the effects Cd(II) may exert on kidney function [9].

Various studies were set out to detect Cd(II), some of them are presented here; such as atomic absorption spectrometry [10–15], X-ray fluorescence spectrometry [16, 17], and mass spectrometry with inductively coupled plasma(ICP-MS) [20–23].

In recent years, veritable growing attention has been directed toward voltammetric techniques as powerful and established methods for the analysis of trace cadmium ions in contaminated foodstuffs and biological samples. Among those methods, differential pulse anodic stripping voltammetry (DPASV) [21–27], linear sweep voltammetry [28, 29], adsorptive stripping voltammetry [30–34] and square-wave voltammetry (SWV) [35–40].

To our knowledge, there is no study of using waste materials in nanoparticles size for modification of carbon paste electrode. So, the big challenge in our study is to obtain a low-cost adsorbent with a high surface area through the development of nanoparticles of mango seed kernel (MSK-NPs). Moreover, the developed adsorbent was used as a modifier of graphite reinforcement carbon paste electrode. (GRCPE) for electrochemical determination of Cd(II) in real samples by differential pulse adsorptive anodic stripping voltammetric method (DPA_d_ASV).

## Experimental

### Reagents and chemicals

All reagents/chemicals were of commercial (analytical) grade and were used as received without any further purification unless otherwise specified. Powder mechanical pencil-lead 60 mm was purchased from [ceramics-XQ (China)]. Paraffin oil [El-Motahda (Egyptian)] used for synthesis of CP. All chemicals used for preparation of buffers and tested solutions were of analytical grade [Merck (Germany) or Sigma-Aldrich (UK)]. Standard solutions of Cd(II) were prepared by dissolving appropriate amount of CdCl_2_.H_2_O in doubly distilled water (DDW).

All other standard solutions such as Co(NO_3_)_2_.6H_2_O, Pb (NO_3_)_2_, and FeCl_3_ as interfering ions were used for studying the selectivity. Five types of buffer solutions such as Britton-Robinson (B.R.), phosphate (KH_2_PO_4_/HCl), chloride (KCl/HCl), hydrochloric (HCl/ KCl) and phthalate (‏C_8_H_5_KO_4_/HCl) were used.

### Preparation of bare carbon paste electrode

The bare graphite reinforcement carbon paste electrode (BGRCPE) was prepared by hand-mixing 70% (*w*/*w*) powdered mechanical pencil-leads (60 mm) with 30% (*w*/*w*) mineral paraffin oil in a mortar and pestle until a completely homogeneous stiff paste was obtained. The homogenized carbon paste was allowed to rest for a minimum period of 24 hrs [40]. Then the carbon paste (CP) was packed and pressed firmly into the 2.0 mm internal diameter and 3.3 cm long tip of Micropipette, to produce the BGRCPE.

The surface of the electrode was polished on weighing paper until shine surface was obtained, and a copper wire was introduced from the back side of the tip to provide electrical communication. The BGRCPE was immersed into a glass cell containing supporting electrolyte, and several runs were applied to obtain a low background current as reported elsewhere [41].

### Development of nanocomposite graphite reinforcement carbon paste electrode

#### Preparation of nanoparticles mango seed kernels

The seeds of mango fruit (four samples) were removed manually using stainless-steel knives and were opened to get kernels. The kernels were washed with DDW and air dried. The dried material was ground in a mill and sieved to obtain a fine powdery form and remove the large particles. The powder was also ground and sieved another time. The final powdered material was washed with DDW and dried well in a drying oven at 60 °C for 1 h. Then, it was kept in a closed dark glass container and stored until utilization. The particle size of the powdered kernels records ranges from 11.0 to 17.0 nm using transition electron microscopy [42].

#### Preparation of mango seed kernels nanoparticles/graphite reinforcement carbon paste electrode

The nanocomposite MSK-NPs@GRCPE was prepared by mixing 0.73 g of finely powdered graphite leads with 0.07 g of MSK-NPs, as an optimal dose selected for the modification, by milling in a mortar and pestle. Then, 0.2 g of paraffin oil was added and milled again to give a homogeneous stiff MSK-NPs@GRCPE. A portion of the paste approximately 0.0807 g was packed and pressed firmly into a plastic tip of micropipette with the inner diameter of 1 mm in the front side and 6.5 mm in the back side and long of 4.25 cm to prepare MSK-NPs@GRCPE.

A copper wire, with a diameter of 2 mm and allowing a current ranging from 12 to 220 V to pass through it, was introduced from the back side of the tip to provide electrical connection. The shine surface of the modified electrode was obtained by polishing on weighing paper and rinsing with DDW.

### Preparation of supporting electrolyte (Britton-Robinson buffer)

B.R. buffer (pH 2:6) was prepared through mixing appropriate volumes of acidic and basic buffer components. The acidic buffer mixture comprises 0.04 M boric acid (H_3_BO_3_), 0.04 M glacial acetic acid (CH_3_COOH) and 0.04 M orthophosphoric acid (H_3_PO_4_). The basic buffer component is 0.2 M sodium hydroxide solution (NaOH). Supporting electrolytes were made by adding 10.0 mL of the B.R. buffer of appropriate pH in the electrochemical cell.

### Preparation of standard solution

1.0 × 10^–3^ M of the investigated substance Cd(Cl)2.H2O was prepared from stock solution of 0.1 M. All working standards for calibration were prepared by diluting the primary stock solution with DDW.

### Sample collection and preparation

#### Chocolate sample

Galaxy Chocolate bare were purchased from the local market. 20.0 g of tablet chocolate sample was ashed in quartz crucible for 4 hrs. On a hot plate, and the charred material was transferred to a furnace for overnight heating at 450 °C. The residue was cooled and treated with 10.0 mL concentrated nitric acid and 3.0 mL 30% H_2_O_2_ then kept in the furnace for 2 hrs. At the same temperature so that no organic compound traces are left. The final residue was treated with 0.5 mL concentrated hydrochloric acid and 1.0 – 2.0 mL 70%perchloric acid and evaporated to fumes so that all the cadmium metal changes to cadmium ions [43].

#### Preparation of Egyptian white rice sample

White Egyptian rice was purchased from the local market. 20.0 g of Egyptian white rice sample was accurately weighed in a quartz crucible. 10.0 mL of concentrated sulfuric acid was added and evaporated to near dryness; then 10.0 mL of nitric acid 1:1 (*v*/*v*%) was added and evaporated to dryness. To the previous, concentrated hydrogen peroxide was added drop by drop until the solution gets clear and evaporated to dryness. DDW was added for washing to remove the excess hydrogen peroxide. The residue was then cooled, transferred into a 50 mL volumetric flask, and diluted with DDW [44].

### Apparatus

The content of Cd(II) was detected through electrochemical measurement which was carried out on MSK-NPs@GRCPE using TRACE ANALYSER (AMEL “Italy”) model: 433 with a conventional electrochemical cell containing a three electrode system. The modified graphite reinforcement carbon paste electrode, previously described, served as a working electrode, an Ag/AgCl electrode filled with saturated KCl as a reference electrode in respect to which all measured potentials were given, and a platinum wire as a counter electrode. A magnetic stirrer and stirring bar supported the convective transport during pre-concentration. The peak heights were automatically measured using the ‘tangent fit’ capability of the instrument.The pH measurements were achieved using pH meter (Fisher Scientific Accumet^®^ “USA”) model: 825 which was calibrated with standard buffer solution (pH 4.01 and pH 7.01).

Scanning electron microscopy (SEM) images that illustrate the surfaces’ morphology of different synthesized electrodes were gotten by Scanning electron microscope (JEOL “Japan”) model: JSM ‒ 5400 LV. The particle size and surface morphology of the MSK were characterized using transition electron microscopy (JEM100CX11 JEOL, Japan).

Fourier transform infrared (FTIR) spectra of the materials were recorded for graphite, graphite reinforcement, and MSK-NPs over the range of 400‒4000 cm^−1^ on FTIR (Thermo scientific "USA") model: Nicollet IS10 with 16 cm^−1^ resolution, using the potassium bromide disk method. All experimental measurements were performed at room temperature (25 ± 1 °C).

### Experimental measurements

The fresh surface of MSK-NPs@GRCPE is first electrically activated by five replicated direct current sweeping by the manner of DPA_d_ASV from –1500 to 1500 mV with a scan rate of 100 mVs^−1^, by immersing in 10.0 mL BR buffer of pH 3.9 in the electrochemical vessel. After the voltammogram of buffer had been recorded, aliquots of the Cd(II) standard were added by micropipette and the differential-pulse adsorptive anodic stripping voltammogram was repeated at the same electrode surface under the optimum voltammetric conditions. All experiments were done at room temperature (25 ± 1 °C).

## Results and discussion

### Surface characteristics of the mango seed kernels nanoparticles

The preliminary assessment of the characteristics of mango seed kernels surface groups was necessary to study those seeds as adsorbents. This had a vital role to evaluate the major groups that may contribute to the adsorption, regardless what mechanisms were involved.

The FTIR spectrum gave some valuable information about the presence of adsorbent groups in MSK-NPs; which support the relation between cadmium adsorption and the idea of GRCPE 'modification using powder of these kernels. The surface groups of mango seed may have acidic (carboxylic acids, carboxy lactone, carbonyl, and phenolic) and basic (pyrone and chromen) characteristics [44].

**Fig. 1:**
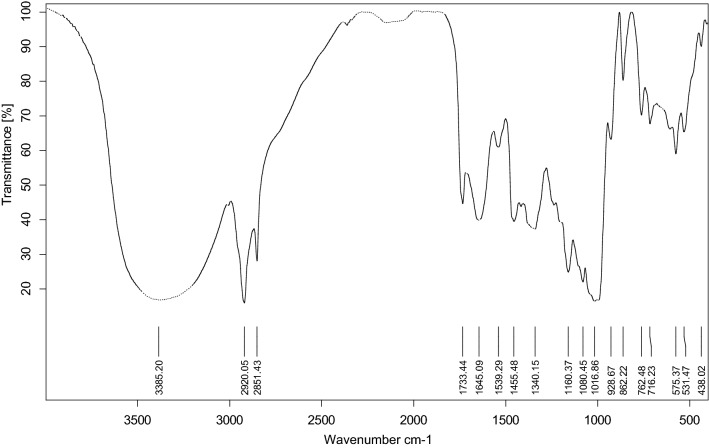
FTIR spectrum of mango seed kernel nanoparticles

In this section, mango seed kernels nanoparticles (MSK-NPs) were characterized using FTIR spectrum Fig. 1. When analyzing the main assignment of bands from the FTIR spectrum Table 1 signal of ester of a carboxylic acid was observed. The region between 1750 and 1600 cm^−1^ was characteristic of carbonyl group. An intense band at ≈ 1733.44 cm^−1^ in MSK-NPs was an indicative of a C = O group from ester of carboxylic acid.

**Table 1:** Assignment of bands in FTIR spectrum of the mango seed kernels nanoparticles

FTIR bands (cm^−1^)	FTIR assignment
1733.44	C=O of carboxylic acids and ester vibration
1645.09	C=O of carbonyl groups vibration
1539.29	C=C aromatic ring stretching
1455.48	Methylene C–H bending
1340.15	Methyl C–H bending
1160.37	C–O–C of ester vibration
1080.45	C–O–C of ester vibration
1016.86	C–O vibration

There is a high amount of surface phenolic groups in MSK-NPs which showed a band in the FTIR spectrum at ≈ 1539.29 cm^−1^. This band is attributed to C = C stretching of the phenolic group. Moreover, phenolic and carbonyl groups are responsible for much of adsorption of heavy metals, and these are the prevailing functional groups in most adsorbents [44]. The enrichment with these groups provides a hydrophilic character to the adsorbent, facilitating their interaction with adsorbate. Now we can say that, MSK-NPs are an important part of mango that has a superior capacity to adsorb Cd(II) from aqueous solutions.

### Morphological characterization of the electrode

**Fig. 2:**
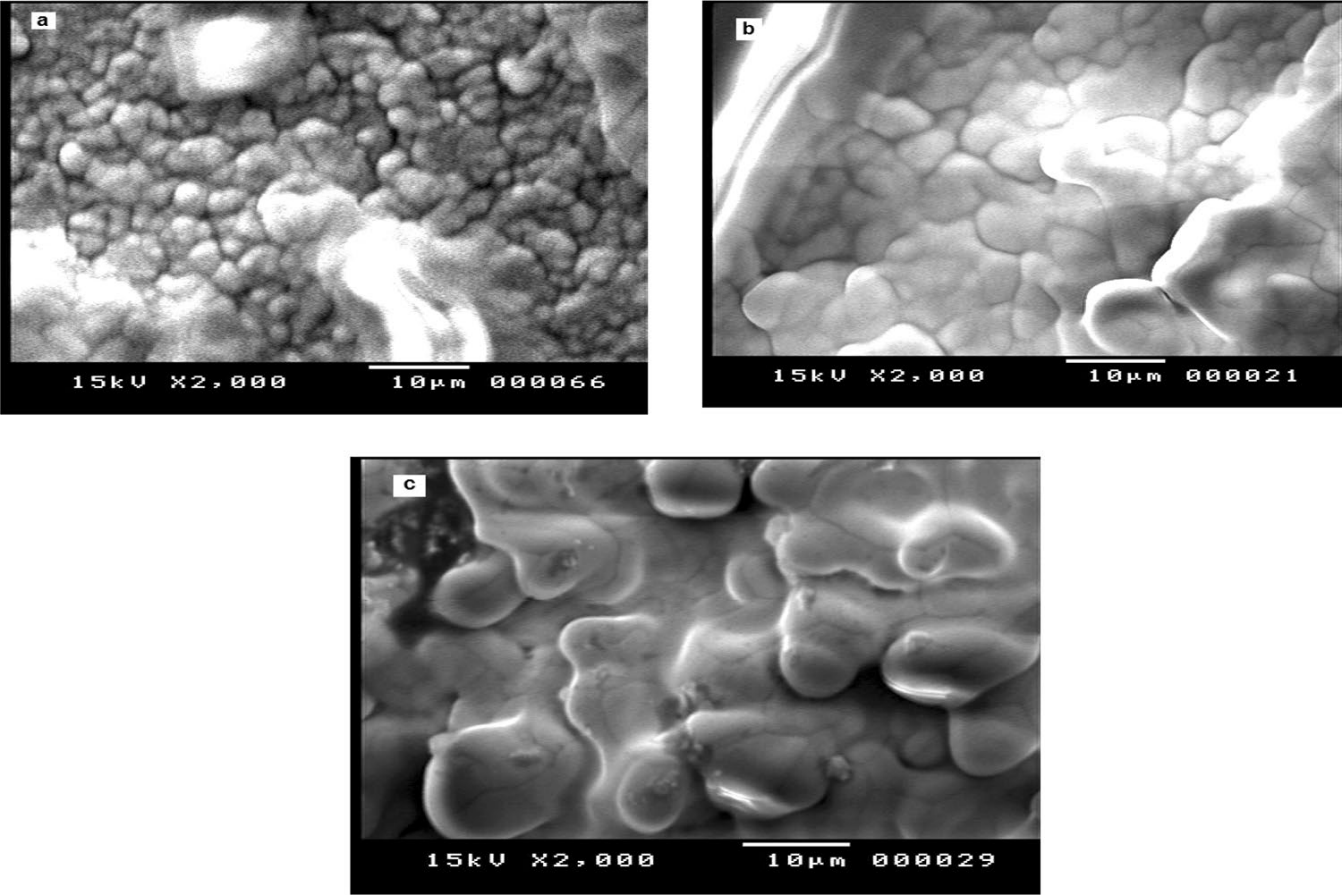
SEM micrographs of (**a**) BGRCPE, (**b**) MSK-NPs@GRCPE, and (**c**) Cd(II) at MSK-NPs@GRCPE

Scanning electron microscopy (SEM) was used to obtain information on the surface morphology of BGRCPE, Fig. 2 a, MSK-NPs@GRCPE, Fig. 2b, and Cd(II) at MSK-NPs@GRCPE, Fig. 2c. The BGRCPE surface was compact and rough, whereas the morphology of MSK-NPs@GRCPE displayed a smooth surface for the electrode The smooth surface of MSK-NPs@GRCPE generated a large surface structure to accommodate Cd(II). Moreover, the porous surface of MSK-NPs@GRCPE enhanced the sensitivity for the detection of Cd(II) in the light of the adsorptive functional group that is present on the surface of MSK-NPs. The different morphological structure shown in Fig. 2c confirms the adsorption of Cd(II) that results in increasing the surface smoothness.

### Electrochemical behavior of cadmium (II)

**Fig. 3:**
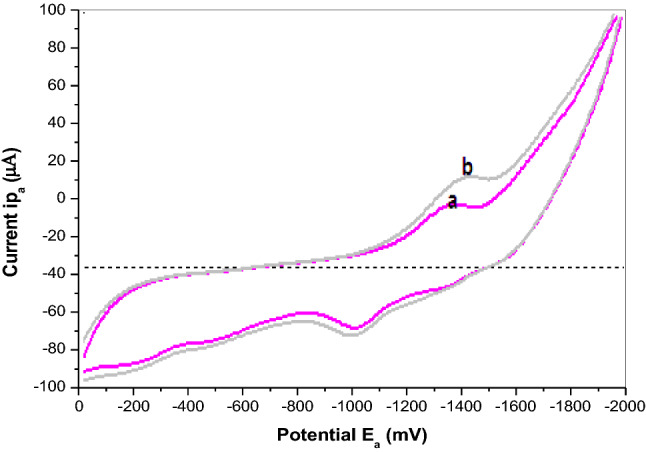
Cyclic voltammograms obtained for 5.0 × 10^–7^ M Cd(II) at (**a**) BGRCPE, and (**b**) MSK-NPs@GRCPE in BR buffer pH 3.9 at scan rate 50 mV s^‒1^, equilibrium time = 15 s

**Scheme 1:**
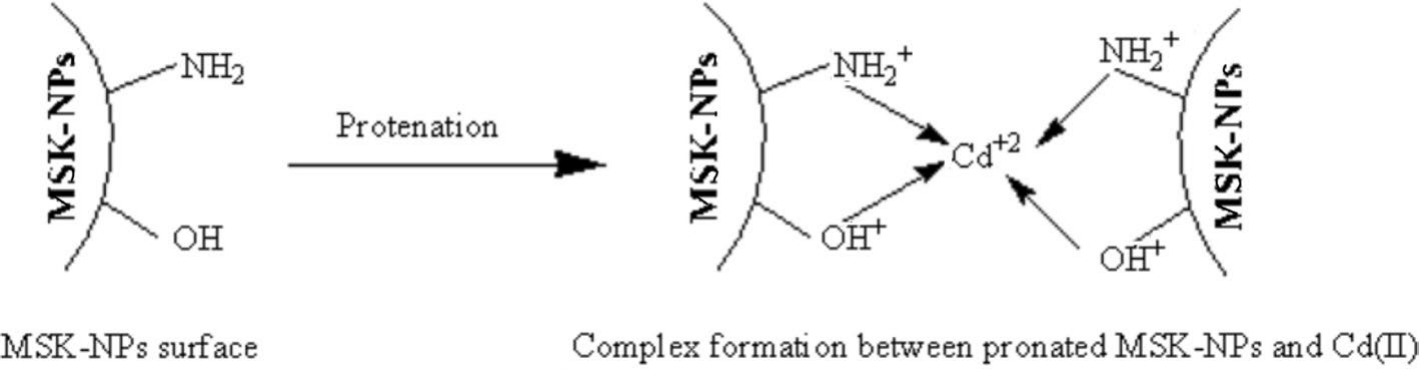
Suggested mechanism of Oxidation of MSK-NPs in acidic medium (electrochemical modification) followed by the complex formation between pronated MSK-NPs and Cd (II)

Figure 3 shows cyclic voltammograms (CVs) of 5.0 × 10^–7^ M Cd(II) in 10.0 mL BR buffer pH 3.9 at different electrodes: (a) BGRCPE and (b) MSK-NPs@GRCPE. It can be seen that Cd(II) showed reversible oxidation reaction accompanied with a pair of well-defined redox peaks at both BGRCPE and MSK-NPs@GRCPE. The adsorption of Cd(II) on the surface of MSK-NPs facilitates the electrochemical oxidation of Cd(II) at MSK-NPs@GRCPE. As also can be seen that at the BGRCPE, Cd(II) was reversibly oxidized with an anodic peak potential at around (‒ 1.350 V) as the oxidation of MSK-NPs occurs first at BGRCPE, then complexation process between protonated groups on MSK-NPs@GRCPE surface and Cd(II) occurred. This makes the oxidation peak of Cd(II) recoverable, which is a highly beneficial property as described in the suggested mechanism, Scheme 1. While the anodic peak potential of Cd(II) at MSK-NPs@GRCPE (‒ 1.430 V) shifted more negatively than that at the BGRCPE, suggesting that MSK-NPs could reduce the overpotential of Cd(II) and accelerate the electron transfer rate, which shows that MSK-NPs may have subtle electronic characteristics. Moreover, the redox peak currents of Cd(II) on MSK-NPs@GRCPE were stronger than on BGRCPE. These results reflect the electrocatalytic activity of MSK-NPs toward Cd(II), and the synergic effect of them as co-catalyst which made the MSK-NPs@GRCPE show higher electrocatalytic activity than BGRCPE.

**Fig. 4:**
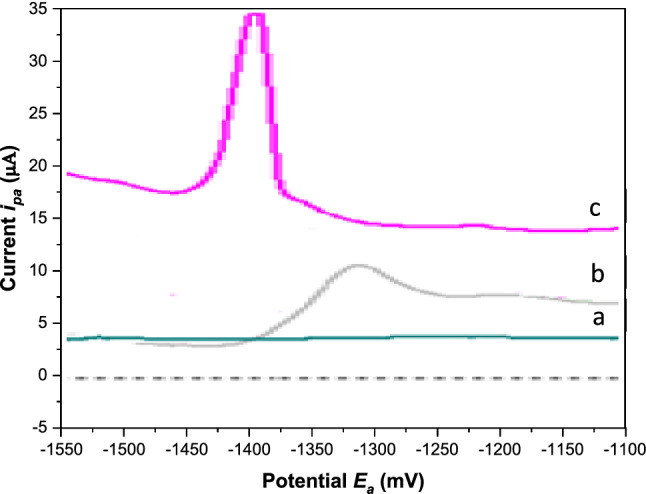
DPA_d_AS Voltammograms for (**a**) Blank; (**b**) 5.0 × 10^–7^ M Cd(II) at BGRCPE; (**c**) 5.0 × 10^–7^ M Cd(II) at MSK-NPs@GRCPE in BR buffer pH 3.9 at *E*_*acc*_ = 1400 mV, *t*_acc_ = 30 s, pulse width = 10 ms, sampling time = 8 ms, pulse repetition = 50 ms and pulse amplitude = 50 mV

Figure 4 represents the differential-pulse anodic peaks of (a) buffer at BGRCPE, (b) 5.0 × 10^–7^ M Cd(II) at BGRCPE, and (c) 5.0 × 10^–7^ M Cd(II) at MSK-NPs@GRCPE. Comparison between Fig. 4b, c implies that MSK-NPs enhanced the electrochemical oxidation of Cd(II) dramatically, thereby accounting for the observed enhanced response.

### Differential-pulse adsorptive anodic stripping voltammetry

#### Effect of pH and supporting electrolyte as experimental conditions

**Fig. 5:**
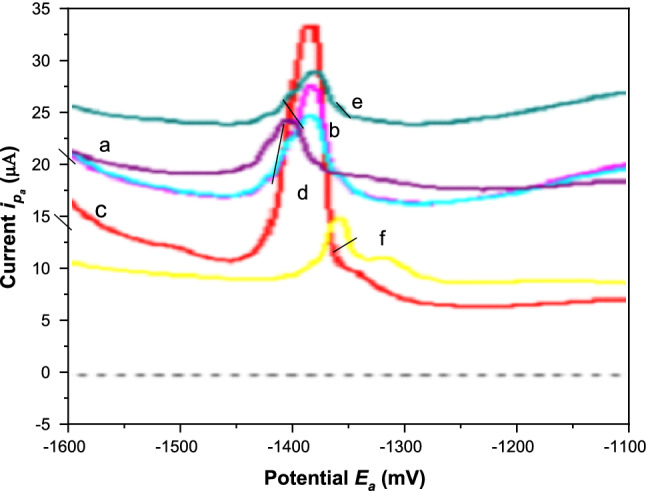
DPA_d_AS voltammetric peak current (*i*_pa_), for 5.0 × 10^–7^ M Cd(II) at different pHs of BR buffer, at *E*_acc_ = 1400 mV, *t*_acc_ = 30 s, pulse width = 10 ms, sampling time = 8 ms, pulse repetition = 50 ms and pulse amplitude = 50 mV. at pH^,^s (**a**) 1.81, (**b**) 2.9, (**c**) 3.9, (**d**) 5.02, (**e**) 7.0, (**f**) 10.0

**Fig. 6:**
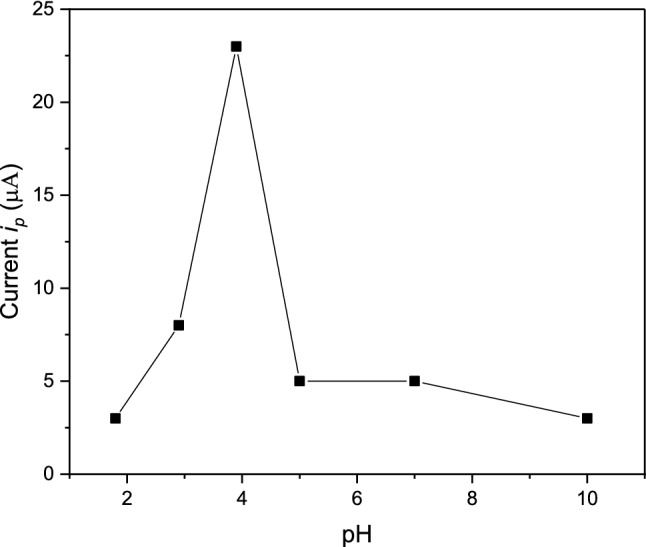
Effect of pH on DPA_d_AS voltammetric peak current (*i*_pa_), for 5.0 × 10^–7^ M Cd(II) at different pHs of BR buffer, at *E*_acc_ = 1400 mV, *t*_acc_ = 30 s, pulse width = 10 ms, sampling time = 8 ms, pulse repetition = 50 ms and pulse amplitude = 50 mV

The influence of the pH of the buffer solution ranging from 1.81 to 10.0 on the electrochemical behavior of Cd(II) was evaluated Fig. 5. As shown in Fig. 6, the oxidation peak currents of Cd(II) increased sharply in the pH range 1.81–3.9 and then decreased slowly in the pH range 3.9–10.0. At pH 3.9, maximum pre-concentration of Cd(II) and consequently maximum current responses were observed. Therefore, pH 3.9 was chosen as an optimum pH for further studies.

**Fig. 7:**
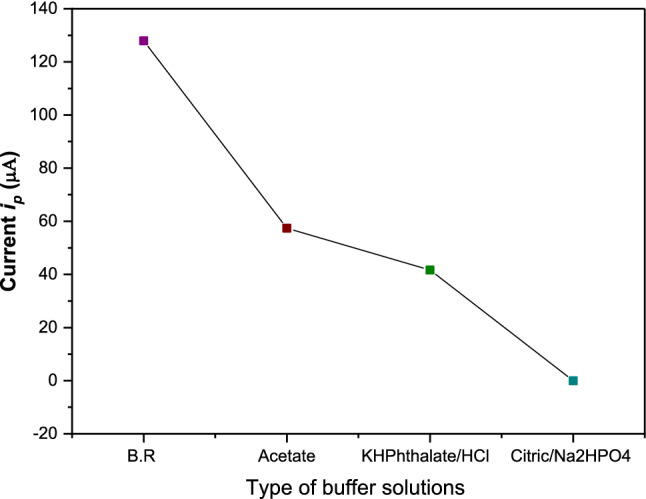
Effect of different buffers on DPA_d_AS voltammetric peak current (*i*_pa_), for 5.0×10^–7^ M Cd(II) at pH ≈ 4.0, at *E*_acc_ = 1400 mV, *t*_acc_ = 30 s, pulse width = 10 ms, sampling time = 8 ms, pulse repetition = 50 ms and pulse amplitude = 50 mV

The voltammetric response is mainly dependent on the pH of the buffer. So electrochemical measurements are usually carried out in a variety of supporting electrolytes to reduce the effect of migration, decrease the resistance of the solution, and keep a constant ionic strength. Different supporting electrolytes such as BR pH 3.9, acetic-acetate buffer pH 4.0, citro-Phosphate pH 4.0 and, acid phthalate pH 4.0 were used in choosing the appropriate supporting electrolyte [41, 45, 46]. Results in Fig. 7 showed that the highest peak height was achieved in the BR buffer.

#### Optimization of the operational parameters

To improve the sensitivity for the determination of Cd(II), the influences of parameters of differential pulse voltammetry on the measurement of Cd(II) were investigated.

#### Effect of accumulation potential (*E*_acc_)

**Fig. 8:**
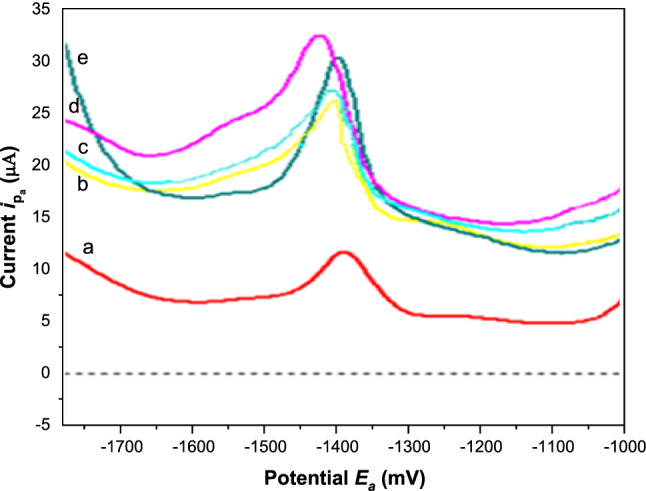
DPA_d_AS voltammetric peak current (*i*_pa_), for 5.0 × 10^–7^ M Cd(II) at different accumulation potentials in BR buffer pH = 3.9, at *t*_acc_ = 30 s, pulse width = 10 ms, sampling time = 8 ms, pulse repetition = 50 ms and pulse amplitude = 50 mV. (**a**) at *E*_acc_ = 800 mV, (**b**) at *E*_acc_ = 1100 mV, (**c**) at *E*_acc_ = 1200 mV, (**d**) at *E*_acc_ = 1300 mV, (**e**) at *E*_acc_ = 1400 mV

**Fig. 9:**
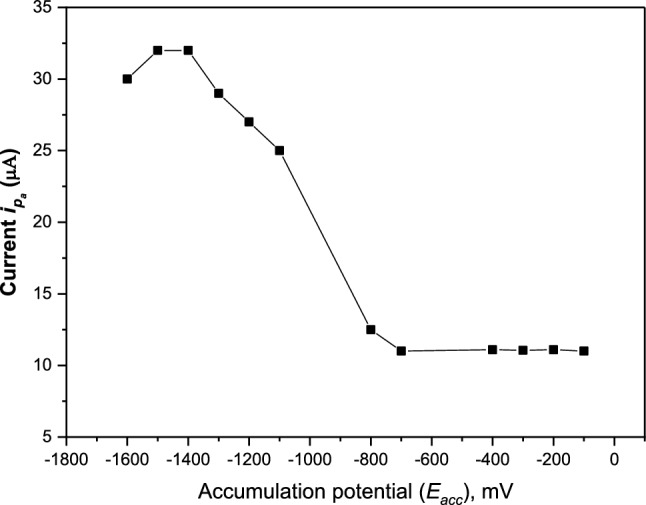
Effect of accumulation potential *E*_acc_ on the adsorption of 5.0 × 10^−7^ M Cd(II) at MSK-NPs@GRCPE in BR buffer pH 3.9

The effect of *E*_acc_ on the differential-pulse voltammetric signal of 5.0 × 10^–7^ M Cd(II) over the range of ‒100 to ‒1600 mV at constant *t*_*acc*_ = 30 s in stirred solution was illustrated in Figures. 8, 9. The obtained results are shown in Fig. 9 which obtained from Fig. 8 revealed that the peak currents of Cd(II) were independent of accumulation potential at the potential range of ‒ 100 to ‒ 700 mV. Whereas the sensitivity of oxidation of Cd(II) increased linearly at potentials whose values were more negative than ‒ 800 mV where at the potential range of ‒ 800 to ‒ 1400 mV. The peak current of Cd(II) was constant between the potential range from ‒ 1400 mV to ‒ 1500 mV, then decreased at ‒1600 mV. This decrease can be attributed to Cd(II) being oxidized immediately without any accumulation; thus, the accumulation efficiency of Cd(II) decreased and resulted in a decrease in the adsorptive response of Cd(II) at the electrode surface. Hence, the accumulation potential *(E*_*acc*_) of ‒ 1400 mV was selected for Cd(II) accumulation due to exhibiting a constant current trend and providing the best sensitivity.

#### Effect of accumulation time (*t*_acc_)

**Fig. 10:**
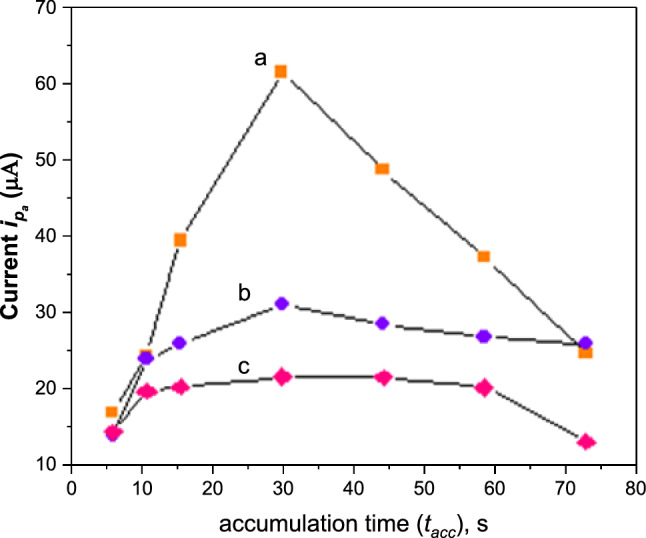
Effect of accumulation time t_acc_ on the adsorption of (**a**) 5.0 × 10^–6^, (**b**) 5.0 × 10^–7^, and (**c**) 5.0 × 10^–8^ M Cd(II)

The influence of varying accumulation time (*t*_acc_) on the current response of Cd(II) at MSK-NPs@GRCPE was examined at three concentration levels 5.0 × 10^–6^ M, 5.0 × 10^–7^ M and 5.0 × 10^–8^ M Cd(II) under different accumulation times from 5 to 75 s at *E*_acc_ = ‒ 1400 mV and the results were summarized in Fig. 10. The anodic peak current increased linearly with *t*_acc_, gradually leveling off at periods longer than 30 s which was presumably due to the saturation of the MSK-NPs@GRCPE surface with Cd(II) at longer *t*_acc_. Thus, 30 s was chosen as optimal *t*_acc_ throughout as it provided relatively high peak current with adequate practical time.

#### Effect of mango seed kernels nanoparticles dosage

**Fig. 11:**
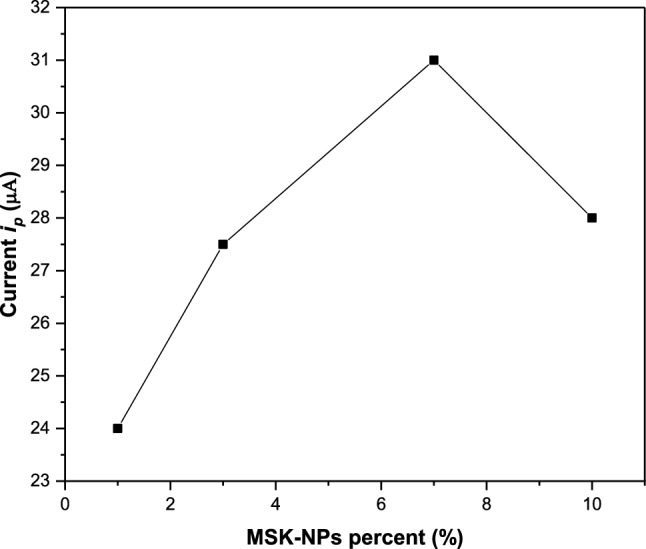
Effect of MSK-NPs dosage on the adsorption of 5.0 × 10^–7^ M Cd(II) at MSK-NPs@GRCPE in BR buffer pH 3.9

Various modified graphite reinforcement carbon pastes containing different weight percent of MSK-NPs 1, 3, 7 and 10% were investigated to study its effect on the response of MSK-NPs@GRCPE to Cd(II). As indicated by Fig. 11, the adsorptive stripping peak current for Cd(II) increased with increasing the percentage of MSK-NPs up to 7% and then decreased. This observation may be due to reducing the graphite reinforcement carbon inherent electrode features with increasing of MSK-NPs more than 7%. Thus 7% of MSK-NPs was selected as the optimum weight present of the modifier.

### Method validation

#### Analytical performance

The optimum parameters selected for voltammetric determination of Cd(II) have been applied to attempt the calibration curve; the recorded results estimate the validity of nanocomposite MSK-NPs@GRCPE in the determination of Cd(II). The linearization equation associated with the calibration curve was *i*_*pa*_ (µA) = 76.27873 + 11.77506 C (M/10^–7^) [r^2^ = 0.99995] which showed a good linear response of Cd(II) at MSK-NPs@GRCPE in the range of 5.0 × 10^–11 ^– 2 × 0.10^–6^ M. These results supported the proposed procedure for determination of Cd(II) using the nanocomposite electrode. The LOD and LOQ of MSK-NPs@GRCPE were calculated at 5.44 × 10^–9^ and 1.65 × 10^–8^ M respectively, Table 2. Compared with BGRCPE, the nanocomposite MSK-NPs@GRCPE has a lower detection limit, indicating that the presence of MSK-NPs could greatly improve the response of BGRPE to Cd(II).

**Table 2:** Characteristic of the calibration plots of Cd(II)^a^ at *E*_*acc*_ = – 1400 mV, pulse width = 10 ms and sampling time = 8 ms

Parameter	*t*_*acc*_ = 30 s
Linearity range (*M*)	5.0 × 10^–11 ^– 2 × 0.10^–6^
Regression equation	*i*_pa_ (µA) = 76.27873 + 11.77506 C (M/10^–7^)
Correlation coefficient (*r*)	0.99997
Determination coefficient (*r*^2^)	0.99995
LOD (M)	5.44 × 10^–9^
LOQ (M)	1.65 × 10^–8^

^a^Average of five determinations

In combination of the strong adsorption ability of MSK-NPs with the metallic and nonmetallic features of GRCPE, the MSK-NPs@GRCPE exhibited excellent performance for the determination of the trace Cd(II).

#### Selectivity and interferences study

Under the optimal experimental conditions, interferences studies were carried out by adding foreign species such as Pb(II), Co(II) and Fe(III) to a fixed concentration of Cd(II). These metal species were used as they might be expected to exhibit redox activity in the same potential range as Cd (II), or compete with Cd (II) for adsorption at the surface of MSK-NPs. The results in Table 3 showed that there was no change in the peak currents of Cd(II), suggesting that MSK-NPs@GRCPE has excellent selectivity.

**Table 3:** Change in peak current of 5.0 × 10^–7^ M Cd(II) in the presence of different metal species

Interfering metal species	Concentration (*M*)	Peak current change (%)
Pb(II)	5.0 × 10^–7^	+ 0.5
Co(II)	5.0 × 10^–7^	− 1.5
Fe(III)	5.0 × 10^–7^	+ 2.5

#### Reproducibility

The analytical precision of our proposed method was estimated from the reproducibility of MSK-NPs@GRCPE for the electrochemical response of Cd(II) that was studied at a concentration level of 5.0 × 10^–7^ M by ten successive measurements with the relative standard deviation (RSD %) of 2.1%, indicating good reproducibility of MSK-NPs@GRCPE.

#### Real sample analysis

**Fig. 12:**
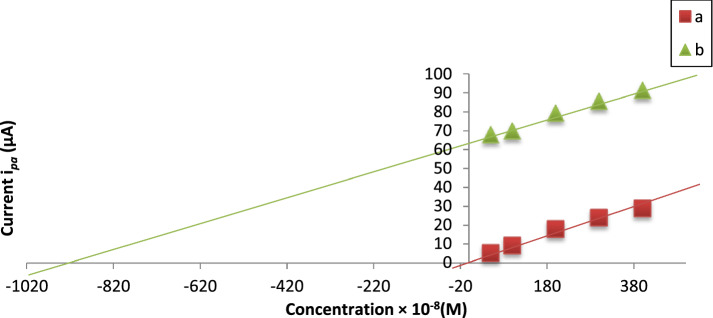
Standard addition plot for Cd(II) determination in chocolate samples. (**a**) Unspiked chocolate sample, (**b**) Spiked chocolate sample

**Fig. 13:**
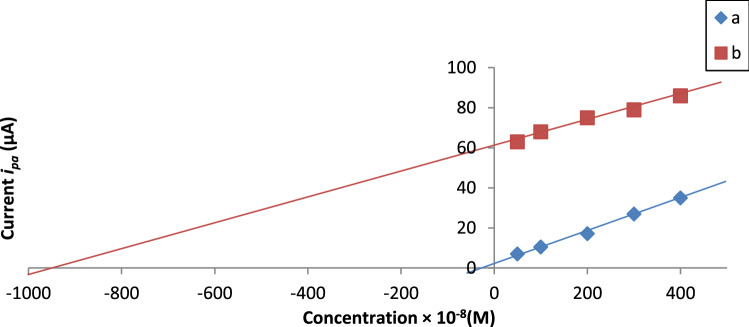
Standard addition plot for Cd(II) determination in Egyptian white rice samples. (**a**) Unspiked Egyptian white rice sample, (**b**) Spiked Egyptian white rice sample

The practical applicability of the electrode was verified by the determination of Cd(II) in chocolate and Egyptian white rice samples were used without spiking and after spiking. The determination of Cd(II) traces was carried out using multiple standard addition techniques to calculate and correct the results of the test samples and then determined by the MSK-NPs@GRCPE according to the previous procedure. The amount of metal traces present in the sample was determined by plotting the standard addition calibration curve between current (*i*_pa_) versus added volumes of standard (*V*_std_). Standard addition calibration curves of un-spiked and spiked chocolate/Egyptian white rice samples which are given in Figures. 12, 13 were straight lines.

The results were summarized in Tables 4, 5; these data revealed that the new developed electrode is suitable for the determination of Cd(II) in real samples.

**Table 4:** Recovery results in chocolate sample

Found values of Cd(II) in unspiked Chocolate sample^a^(M)	Added value of Cd(II) standard solution(M)	Found values of Cd(II) in spiked chocolate sample^a^(M)	Found value of Cd(II) standard solution^a^ (M)
6.88 × 10^–8^	1.0 × 10^–4^	1.010688 × 10^–4^	1.01 × 10^–4^
Mean recovery %	101.0		
RSD %	1.0		

^a^Average of five determinations

**Table 5:** Recovery results in Egyptian white rice sample

Found values of Cd(II) in unspiked Egyptian white rice sample^a^(M)	Added value of Cd(II) standard solution(M)	Found values of Cd(II) in spiked Egyptian white rice sample^a^(M)	Found value of Cd(II) standard solution^a^(M)
24.96 × 10^–8^	1.0 × 10^–4^	9.92496 × 10^–5^	0.99 × 10^–4^
Mean Recovery %	99.0		
RSD %	1.58		

^a^Average of five determinations

## Conclusion

The fabrication of carbon paste electrode with MSK-NPs gives a highly sensitive and selective electrode for voltammetric determination of Cd(II) in real samples. The optimum parameters selected have been applied to attempt the calibration curve; the recorded results estimate the validity of nanocomposite MSK-NPs@GRCPE in the determination of Cd(II). So, this novel electrode can be selected as a reliable tool for the determination of Cd(II) in real samples even if these ions are present at trace levels.
